# Opioid versus non-opioid postoperative pain management in otolaryngology

**DOI:** 10.1186/s12871-023-02213-x

**Published:** 2023-08-25

**Authors:** Allison Keane, Kayla Jardine, David Goldenberg, Sandeep Pradhan, Jay Zhu, Jobran Mansour, Hadas Knoller, Ron Eshel, Yoav P. Talmi, Sonia Vaida, Guy Slonimsky

**Affiliations:** 1 Department of Otolaryngology-Head and Neck Surgery, Penn State Health, Milton S. Hershey Medical Center, 500 University Drive, Hershey, PA 17033 USA; 2 The Pennsylvania State University, College of Medicine, Hershey, PA USA; 3 Department of Public Health Sciences, The Pennsylvania State University, College of Medicine, Hershey, PA USA; 4 Department of Otolaryngology-Head and Neck Surgery, the Chaim Sheba Medical Center, Tel Hashomer, Israel; 5 Division of Anesthesia, Intensive Care, and Pain Management, Tel-Aviv Medical Center, Tel-Aviv University, Tel-Aviv, Israel; 6 Department of Anesthesiology and Perioperative Medicine, Penn State Health, Milton S. Hershey Medical Center, Hershey, PA USA

**Keywords:** Pain management, Postoperative, Opioid, Otolaryngology

## Abstract

**Background:**

The opioid epidemic in the United States has had devastating consequences, with many opioid-related deaths and a significant economic toll. Opioids have a significant role in postoperative pain management. Here we aim to analyze differences in postoperative opioid and non-opioid pain medications regimens following common otolaryngological surgeries between two large tertiary care medical centers: the Milton S. Hershey Medical Center, USA (HMC) and The Chaim Sheba Medical center, Israel (SMC).

**Methods:**

A retrospective chart review of patients undergoing common otolaryngological procedures during the years 2017–2019 was conducted at two tertiary care centers, one in the U.S. and the other in Israel. Types and doses of postoperative pain medications ordered and administered during admission were analyzed. Average doses ordered and administered in 24 h were calculated. Opioid medications were converted to a standardized unit of morphine milliequivalents (MME). Chi-square test and Wilcoxon rank-sum test were used to compare the groups.

**Results:**

The study included 204 patients (103 U.S., 101 Israel). Patient demographics were similar except for a longer length of stay in Israel (*p* < 0.01). In the U.S., 95% of patients were ordered opioids compared to 70% in Israel (*P* < 0.01). In the U.S., 68.9% of patients ordered opioids received the medications compared to 29.7% in Israel. The median opioid dose ordered in the U.S. was 45MME/24 h compared to 30MME/24 h in Israel (*P* < 0.01), while median dose received in the U.S. was 15MME/24 h compared to 3.8MME/24 h in Israel (*P* < 0.01). Opioid prescriptions at discharge were given to 92% of patients in the U.S. compared to 4% of patients in Israel (*p* < 0.01). A significantly higher number of patients in the U.S. were prescribed acetaminophen and ibuprofen (*p* < 0.0001). Dipyrone was prescribed to 78% of patients in Israel.

**Conclusions:**

HMC demonstrated a significantly more permissive approach to both prescribing and consuming opioid medications for postoperative pain management than SMC for similar, common otolaryngological surgeries. Non-opioid alternatives and examining the cultural and medical practice-based differences contributing to the opioid epidemic should be discussed and reevaluated.

## Introduction

The opioid epidemic in the United States has had devastating consequences, with many opioid-related deaths and a significant economic toll. According to the CDC, between 1999 and 2010, there was a fourfold increase in opioid prescriptions in the United States [1]. The incidence of deaths involving overdose of prescribed opioids was five times higher in 2016 than in 1999, reaching 42,000 cases [2]. In 2019, prescription opioids accounted for more than 25% of deaths caused by opioid overdose [3]. Along with increased healthcare costs, opioid misuse and abuse is associated with significant collateral costs derived from criminal justice, loss of productivity, and other social consequences [4].

The rate of opioid prescribing by surgeons (36.5%), was found to be second only to the specialties of pain medicine (48.6%) [5]. Hydrocodone and oxycodone containing medications represent the most commonly prescribed formulations in the United States [6, 7]. Many patients do not practice safe disposal of non-consumed medications, which may eventually contribute to opioid abuse, addiction, and death [8, 9]. Moreover, opioid-naive patients are at risk of becoming chronic opioid users following low-risk surgeries [10]. While opioids are considered effective pain relievers when used as indicated and as prescribed, the incidence of opioid abuse, along with the associated morbidity, mortality, and financial costs, makes their long-term usefulness questionable [11, 12].

Data comparing opioid prescription patterns in the United States with the rest of the world is scarce but valuable for self-evaluating post-surgical pain management practices. Other countries, such as Israel, do not seem to be significantly affected by the prescription opioid epidemic. In Israel, the consumption of five potent opioids (requiring a special prescription form) increased by 47% from 2000 to 2008, which is modest compared to the United States [13, 14].

Alternative, non-opioid pharmacological agents can provide adequate pain relief and reduce exposure to opioids.

Dipyrone, also named Metamizole (noramidopyrine‐methanesulfonate), is an analgesic, spasmolytic, and antipyretic commonly prescribed in Israel, parts of Europe, Asia, and South America [15–19]. The mechanism of action of dipyrone is not entirely known and involves cyclooxygenase 1 and 2 inhibition [20]. Dipyrone is associated with a rare risk of agranulocytosis and has been banned in some parts of the world, including the United States, since the 1970s [21–23].

Our study analyzed the differences in the postoperative prescription and the administration of opioid and non-opioid pain medications in adults following common otolaryngological surgeries between two medical centers in the United States and Israel.

## Materials and methods

### Study design and participants

The study was approved by the Penn State Hershey Medical Center Institutional Review Board. Eligible patients were identified via the electronic medical record systems at Penn State Hershey Medical Center (HMC), Hershey, PA. Initial patient query included otolaryngology patients who underwent tonsillectomy, tympanomastoidectomy, thyroidectomy, functional endoscopic sinus surgery (FESS), or neck dissection between the years 2017–2019. Data for the 30 most recent patients that underwent each procedure was collected. A similar query was conducted at the Sheba Medical Center (SMC), Israel. The principal investigator reviewed the procedures to confirm the patients at each institution underwent comparable procedures, and then the data was combined for analysis. Due to differences in medication dosing documentation for pediatric patients between the United States and Israel, all patients under 18 years of age were excluded. Patients with a hospital stay greater than seven days were also excluded.

### Retrospective chart review

Electronic medical records were reviewed at the two institutions for data collection. Patient demographic information, including age, sex, and length of hospital stay, was collected. In addition, data regarding postoperative prescribed pain medications were reviewed. The type and dose of postoperative pain medications ordered and given (medications that confirmed as administered by the nursing team) to patients during admission were recorded, and the average dose ordered and administered in 24 h was calculated. In addition, data regarding the number of patients receiving opioid prescriptions at discharge were collected.

Both scheduled medications and PRN (Pro re nata/as needed) medications were recorded. Orders for both intravenous (IV) and oral opioid and non-opioid medications were recorded. Dosing of opioid medications was converted to a standardized unit of Morphine Milliequivalents (MME) using a conversion calculator available at the Centers for Disease Control and Prevention (CDC) website [24].

Twenty-four hours total was calculated for scheduled medications based on the cumulative dose prescribed within 24 h. For PRN medications, the maximum dose available to a patient during 24 h was recorded. For example, for oxycodone, many patients were prescribed doses of 5 mg and 10 mg PRN based on pain severity. Therefore, the maximum dose of 10 mg PRN was used to calculate the 24-h total available dose to the patient, in case he requested it.

### Statistical analysis

Patient demographic information was compared using a Wilcoxon rank-sum test for age and length of stay, while a Chi-square test was used to compare patient sex and type of surgery. A Chi-square test was used to compare the percentage of patients from each country who were prescribed and received acetaminophen, dipyrone, non-steroidal anti-inflammatory drugs (NSAIDs), and opioids postoperatively. A Chi-square test was also used to compare the percentage of patients that were prescribed opioids upon discharge. A Wilcoxon rank-sum test was used to compare the median dose of medications ordered and received in the hospital. A *p*-value < 0.05 was considered significant.

## Results

### Patient characteristics

Following the exclusion of patients under 18 years of age and patients with a hospital stay greater than 7 days, two hundred and four patients met inclusion criteria and were included in the study, 103 from the United States (HMC) and 101 from Israel (SMC). Of these patients, 106 (52%) were females, and 98 (48%) were males. There was a similar male-to-female distribution between countries (Table 1). The average age of patients at SMC was 50.4 with a median of 51.9 (range 18 – 87), similar to the age of patients at HMC, as shown by a Wilcoxon rank-sum test, *P*-value = 0.0844 (Table 1). Following the exclusion of patients according to the exclusion criteria, the proportion of procedures remained similar between the two medical centers (Table 1). However, the length of stay was statistically different as shown by a Wilcoxon rank-sum test (*P*-value < 0.0001) with a more extended hospital stay observed at SMC.

**Table 1:** Patients demographics

	Country			
	Israel (*N* = 101)	US. (*N* = 103)	Total (*N* = 204)	*P*-value
**Age (years)**				0.0844^2^
Mean (SD)	50.4 (19.05)	46.4 (16.33)	48.4 (17.80)	
Median	51.9	45.4	48.9	
Range	18.0, 87.0	18.2, 88.2	18.0, 88.2	
**Gender**, N (%)				0.3293^1^
Female	49 (48.5%)	57 (55.3%)	106 (52.0%)	
Male	52 (51.5%)	46 (44.7%)	98 (48.0%)	
**Types of Surgery**, N (%)				0.5245^1^
Tonsillectomy	2 (2.0%)	1 (1.0%)	3 (1.5%)	
Tympanomastoidectomy	19 (18.8%)	25 (24.3%)	44 (21.6%)	
Thyroidectomy	30 (29.7%)	29 (28.2%)	59 (28.9%)	
FESS	28 (27.7%)	20 (19.4%)	48 (23.5%)	
Neck dissection	22 (21.8%)	28 (27.2%)	50 (24.5%)	
**Length of Stay (days)**				** < .0001**^**2**^
Mean (SD)	3.0 (1.30)	1.8 (1.07)	2.4 (1.32)	
Median	2.0	2.0	2.0	
Range	2.0, 7.0	1.0, 7.0	1.0, 7.0	

Data is presented as total number of patients (N) and percentage (%)

^1^Chi-Square *p*-value

^2^Wilcoxon rank-sum *p*-value

The prescribed drugs included oxycodone, hydrocodone, hydromorphone, tramadol, morphine, transdermal fentanyl patches, acetaminophen, ibuprofen, and dipyrone (Israel only).

### Non-opioid pain medications

The non-opioid pain medications prescribed by the otolaryngology physicians and received by patients during hospital admission are presented in Table 2.

**Table 2:** Non-opioid medication prescribed and administered in Hospital by Country

	Country			
	Israel (*N* = 101)	US. (*N* = 103)	Total (*N* = 204)	*P*-value
**Acetaminophen**				
**Patients Prescribed in Hospital**, N (%)	8 (7.9%)	94 (91.3%)	102 (50.0%)	** < .0001**^**1**^
**Dose Prescribed in Hospital,** mg/24h				
Mean (SD)	2912.5 (1056.19)	3555.9 (613.71)	3505.4 (674.05)	**0.0128**^**2**^
Median	3000	3900	3900	
Range	500, 3900	1950, 3900	500, 3900	
**Patients Receiving Acetaminophen in Hospital**, N (%)	5 (5%)	70 (68%)	75 (36.8%)	
**Dose Received in Hospital,** mg/24h				
Mean (SD)	653.3 (738.84)	998.3 (508.7)	975.3 (527.54)	0.157^2^
Median	200	975	975	
Range	100, 1800	200, 2507.1	100, 2507.1	
**Ibuprofen**				
**Patients Prescribed in Hospital**, N (%)	2 (2%)	33 (32%)	35 (17.2%)	** < .0001**^**1**^
**Dose Ordered in Hospital,** mg/24h				
Mean (SD)	1975 (35.36)	2412.1 (393.51)	2387.1 (395.44)	**0.0017**^**2**^
Median	1975	2400	2400	
Range	1950, 2000	1200.0, 3600.0	1200, 3600	
**Patients Receiving Ibuprofen in Hospital**, n (%)	0 (0%)	22 (21.4%)	22 (10.8%)	** < .0001**^**1**^
**Dose Received in Hospital,** mg/24h				
Mean (SD)	0	522.7 (220.24)	522.7 (220.24)	
Median	0	600	600	
Range	0	200, 900	200, 900	
**Dipyrone**				
** Patients Prescribed in Hospital**, N (%)	82 (81.2%)	0 (0%)	82 (40.2%)	** < .0001**^**1**^
**Dose Prescribed in Hospital,** mg/24h				
Mean (SD)	2974.4 (585.38)	0	2974.4 (585.38)	
Median	3000	0	3000	
Range	1000, 4000	0	1000, 4000	
**Patients Receiving Dipyrone in Hospital**, N (%)	64 (63.4%)	0 (0%)	64 (31.4%)	** < .0001**^**1**^
**Dose Received in Hospital,** mg/24h				
Mean (SD)	892.6 (732.33)		892.6 (732.33)	
Median	666.7		666.7	
Range	200, 5000		200, 5000	

Data is presented as total number of patients (N) and percentage (%)

^1^Chi-Square *p*-value

^2^Wilcoxon rank-sum *p*-value

Acetaminophen was ordered for 91% of patients at HMC, while only for 7.9% of patients at SMC (*P*-value < 0.0001). The dose of acetaminophen ordered for patients was also significantly higher at HMC with a median of 3900 mg/24 h compared to 3000 mg/24 h at SMC (*P*-value = 0.0128). Sixty eight percent of the patients who were prescribed acetaminophen at HMC eventually received the medication compared to 62.5% at SMC.

Ibuprofen was prescribed for 32% of the patients at HMC, compared to only 2% of patients (a total of 2 patients) at SMC (*P*-value < 0.0001), which eventually did not receive the medication. Of the patients at HMC prescribed ibuprofen, 68.75% received the medication with a median dose of 600 mg/24 h.

The non-opioid medication prescribed and administered most often at SMC was dipyrone, with 81% and 78% of patients having the medication ordered and eventually administered, respectively, with a median dose of 666 mg/24 h.

### Opioid pain medications

The prescribing preferences and administration of opioids between the two medical centers in the U.S. and Israel were significantly different (Table 3). Ninety-five percent of the HMC patients in the study were prescribed opioids postoperatively during their hospital stay compared to 70% of patients at SMC (*P*-value < 0.0001). The dose of opioids ordered was significantly higher at HMC with a median of 45MME/24 h compared to 30MME/24 h at SMC (*P*-value < 0.0001). At HMC, 68.9% of patients prescribed opioids eventually took the medication compared to only 29.7% of SMC patients (*P*-value < 0.0001). Overall, 72% of patients at HMC received opioids, compared to only 42% at SMC. The median dose of opioids received at HMC was 15MME/24 h compared to only 3.8MME/24 h at SMC (*P*-value < 0.0001). Opioids prescriptions at discharge were given to 92.2% of patients at HMC compared to only 4% of patients at SMC (*P*-value < 0.0001).

**Table 3:** Opioid medication prescribed and received in hospital and prescribed at discharge

	Country			
	Israel (*N* = 101)	US. (*N* = 103)	Total (*N* = 204)	*P*-value
**Patients Prescribed Opioids in Hospital**, N (%)	71 (70.3%)	98 (95.1%)	169 (82.8%)	** < .0001**^**1**^
**Opioid dose Pprescribed in Hospital,** MME/24h				** < .0001**^**2**^
Mean (SD)	32.4 (9)	55.1 (20.39)	45.6 (19.98)	
Median	30	45	45	
Range	7.5, 65	22.5, 13	7.5, 135	
**Patients Receiving Opioids in Hospital**, N (%)	30 (29.7%)	71 (68.9%)	101 (49.5%)	** < .0001**^**1**^
**Opioid dose received in Hospital,** MME/24h				** < .0001**^**2**^
Mean (SD)	5.9 (4.94)	15.9 (13.56)	12.9 (12.51)	
Median	3.8	15	7.5	
Range	1.9, 24.4	3.8, 71.3	1.9, 71.3	
**Patients Prescribed Opioids at Discharge**, N (%)	4 (4%)	95 (92.2%)	99 (48.5%)	** < .0001**^**1**^

Data is presented as total number of patients (N) and percentage (%)

*MME*  Morphine milligram equivalents

^1^Chi-Square *p*-value; ^2^ Wilcoxon rank-sum *p*-value

### Pain medication by procedure

The opioid and non-opioid pain medications ordered and administered were analyzed specifically by the surgical procedure (Table 4). Given that only 3 cases of tonsillectomy were identified, data regarding post tonsillectomy pain medication was not included in the analysis.

**Table 4:** Medication prescribed and received in hospital by surgery Type

	Fess			Neck dissection			Thyroidectomy			Tympanomastoidectomy		
	Country			Country			Country			Country		
	Israel (*N* = 28)	US (*N *= 20)	*P*-value	Israel (*N* = 22)	US (*N* = 28)	*P*-value	Israel (*N* = 30)	US (*N* = 29)	*P*-value	Israel (*N* = 19)	US (*N* = 25)	*P*-value
**Opioids**												
**Prescribed in hospital N** (%)	17 (60.7%)	19 (95%)	0.00681	16 (72.7%)	27 (96.4%)	0.0165	23 (76.7%)	26 (89.7%)	0.18371	13 (68.4%)	25 (100.0%)	0.00251
**Received in hospital N** (%)	6 (21.4%)	12 (60%)	0.00651	8 (36.4%)	22 (78.6%)	0.0025	10 (33.3%)	19 (65.5%)	0.01341	4 (21.1%)	18 (72%)	0.00081
**Prescribed at discharge N** (%)	1 (3.6%)	19 (95%)	< .00011	0 (0%)	23 (82.1%)	< .0001^1^	0 (0%)	27 (93.1%)	< .00011	1 (5.3%)	25 (100%)	< .00011
**Acetaminophen**												
**Prescribed in hospital N** (%)	0 (0%)	18 (90%)	< .00011	4 (18.2%)	28 (100%)	< .0001^1^	1 (3.3%)	29 (100%)	< .00011	3 (15.8%)	18 (72.0%)	0.00021
**Received in hospital N** (%)	0 (0%)	10 (50%)	< .00011	3 (13.6%)	25 (89.3%)	< .0001^1^	1 (3.3%)	23 (79.3%)	< .00011	1 (5.3%)	11 (44%)	0.00431
**Ibuprofen**												
**Prescribed in hospital N** (%)	0 (0%)	1 (5%)	0.23181	0 (0%)	6 (21.4%)	0.0206	0 (0%)	25 (86.2%)	< .00011	2 (10.5%)	0 (0%)	0.09681
**Received in hospital N** (%)	0 (%)	0 (%)	-	0 (%)	5 (17.9%)	0.0367	0 (%)	16 (55.2%)	< .00011	0 (0%)	0 (0%)	-
**Dipyrone**												
**Prescribed in hospital N** (%)	23 (82.1%)	0 (0%)	< .00011	16 (72.7%)	0 (0%)	< .0001^1^	26 (86.7%)	0 (0%)	< .00011	15 (78.9%)	0 (0%)	< .00011
**Received in hospital N** (%)	16 (57.1%)	0 (0%)	< .00011	14 (63.6%)	0 (0%)	< .0001^1^	21 (70%)	0 (0%)	< .00011	11 (57.9%)	0 (0%)	< .00011

^1^Chi-Square *p*-value

^2^Wilcoxon rank-sum *p*-value

Data is presented as total number of patients (N) and percentage (%)

*FESS* Functional endoscopic sinus surgery

Except for in hospital opioids prescribed following thyroidectomy, across all surgical procedures analyzed, there were statistically significant differences between the in-hospital prescription and administration of opioids, acetaminophen and ibuprofen, as well as for discharge prescriptions for opioids between HMC and SMC. At HMC, 89.7% of patients undergoing thyroidectomy were prescribed postoperative opioids compared to 76.7% of patients undergoing thyroidectomy at SMC, which was not statistically significant (*P*-value = 0.184). However, at HMC, 65.5% of patients undergoing thyroidectomy eventually received the postoperative opioids compared to only 33.3% at SMC, with statistical significance (*P*-value = 0.013).

Dipyrone was ordered for 72.7–86.7% and administered to 57.1–70% of patients across all analyzed procedures, exclusively at SMC.

Overall, the analysis of postoperative pain medications ordered and administered by procedure was consistent with the findings that opioids, acetaminophen and ibuprofen were prescribed and administered more often at HMC, while dipyrone was ordered and administered exclusively at SMC.

## Discussion

Our study highlights considerable differences in the postoperative prescribing practices and consumption of opioids between two otolaryngology departments in the United States and Israel. This is among the first studies to evaluate the actual in hospital doses of opioids administered following otolaryngological surgeries, to the best of our knowledge. A significantly higher number of HMC patients were prescribed opioids, and more patients eventually consumed them. Moreover, opioid prescriptions at HMC consisted of higher doses, and the patients ultimately consumed higher opioid doses. Ninety-five percent of patients at HMC were prescribed opioids, and 72.4% eventually took them, compared to only 70% and 42.4% at SMC, respectively. Although the in-hospital length of stay at HMC was significantly shorter than at SMC, the overall opioid consumption at SMC was lower. Additionally, upon discharge from the hospital, a substantially greater proportion of HMC patients were prescribed opioids. At HMC, the percentage of patients prescribed opioids in the hospital and at discharge was similar (95% vs 92.2%, respectively). Interestingly, following thyroidectomy, a higher proportion of patients were prescribed opioids at discharge than in the hospital (93.1% vs 89.7%, respectively). As only 68.9% of patients prescribed opioids at HMC eventually received the medication during their hospital stay, overall, the number of patients prescribed opioids at discharge was larger. All these findings, taken together, indicate a more permissive approach towards both prescribing and consuming opioids at HMC and underscore the importance of evaluating the anticipated pain management needs for every patient individually at discharge, specifically for opioids.

The prescription and consumption of acetaminophen and ibuprofen at SMC was also significantly lower compared to HMC. Interestingly, at both HMC and SMC, the administered doses for acetaminophen and ibuprofen, as well as the number of patients receiving the medications, were consistently smaller than prescribed. There are several reasons that potentially account for that finding. Acetaminophen, ibuprofen, and Dipyrone are commonly prescribed as scheduled medications; however, it is possible that for some of the patients these were prescribed PRN, which can result in a smaller dose eventually being administered. Alternatively, it is possible that some patients preferred not to be given doses of scheduled nonopioid pain medications. Another possibility is that patients were discharged before completing the entire 24-h prescribed dose and therefore, the given those was eventually smaller than prescribed. Although these can presumably confound our results, we believe that they affected the patients across our cohorts in a similar fashion both at HMC and SMC.

Dipyrone was prescribed and administered only to SMC patients.

Dipyrone is banned in several countries and available by physician prescription or over the counter in others. Dipyrone was reported to be associated with rare blood dyscrasias, specifically agranulocytosis with a reported incidence ranging between ~ 1:1500 to ~ 1:10^6^ [21–23, 25–32]. It is commonly used in most European Union and Latin America countries and banned in other countries such as the United Kingdom, Sweden, and the U.S. For instance, approval was withdrawn in Sweden in 1974, the U.S. in 1977, and India in 2013, with ban, eventually lifted in India in 2014 [31, 33–35]. Given the widespread, safe adminsitration of Dipyrone outside the U.S., reconsidering its use could broaden the armentarium of non-opiod analgesics, and ultimately reduce opioid prescriptions for these types of surgeries.

In a review of the literature and meta-analysis of randomized controlled trials, with a total of 79 trials including almost 4000 patients, comparing dipyrone to other analgesics, Kotter et al. found no difference in the incidence of short-term adverse events and no reported cases of agranulocytosis [31].

In an overview of Cochrane reviews regarding the efficacy of oral analgesics for acute pain management, Moore et al. reported the highest efficacy for combinations of ibuprofen and acetaminophen. While dipyrone was more effective than acetaminophen as a single agent, it was equally as effective as ibuprofen [36]. Furthermore, significant differences among opioid utilization patterns between the U.S. and other countries were reported in studies that did not include dipyrone [37].

In a recent study, Choo et al. evaluated the difference between the amount of opioids prescribed upon discharge and patient-reported consumption following tonsillectomy at the Ohio State University. The authors found that patients reported a significantly lower amount of opioid consumption than was prescribed to them [38]. Similar results were reported by Pruitt et al. for other common pediatric surgical procedures [39] and Sada et al. for parathyroidectomy [40]. Agamawi et al. found that the utilization of standardized analgesics order sets, of opioid and non-opioid medications, effectively reduced opioids doses dispensed to pediatric patients following tonsillectomy without compromising pain control [41]. The routine over-prescription of opioids and lack of safe disposal may result in excessive narcotics availability to the population.

Kirubalingam et al. found substantial variations in opioids prescribing patterns following otologic surgeries with average MME of 239.73 ± 1097.62, suggesting over prescriptions and demonstrating the need for evaluating safe prescribing practices in order to limit opioid therapy to the lowest effective dose [42].

Cultural factors may also contribute to the permissive prescription and consumption of opioids in the U.S. compared to the rest of the world, combined with outdated misconceptions regarding the efficacy and safety of opioids, fueled by aggressive marketing by the pharmaceutical industry and past Joint Commission erroneous mandates [43–45]. Pain treatment in the acute postoperative period is an important and legitimate goal for surgeons. Poorly managed postoperative pain is associated with lower patient satisfaction, increased physical and mental morbidity, delayed recovery, the risk of chronic pain with prolonged opioid usage, and higher health care costs [46–48].

The concept of multimodal analgesia involves combining opioids with non-opioid agents such as NSAIDs, COX2 inhibitors, local/regional anesthesia etc [49]. It has gained popularity ever since as a practical approach for reducing acute and chronic postoperative pain and opioid consumption [50].

In a recent clinical practice guideline Anne et al. formulated a multimodal treatment plan for opioid prescription following common otolarygological surgeries aiming to reduce the risk of opioids abuse [51]. The authors advocated for non-opioid multimodal analgesia, identifying risk factors for opioids abuse, limiting therapy to the lowest effective dose, pre-operative counseling for pain management, discussing risks associated with opioids and educating for proper disposal of opioids.

Despite the known risks and morbidity associated with their administration, opioids remain the mainstay of postoperative pain management. However, clinicians should always consider available alternatives to opioids or prescribe lower doses of opioids as an adjunct to non-opioid pain medications.

### Study limitations and future perspectives

The data presented here was collected in two large academic medical centers, one in the U.S. and the other in Israel. Although statistically significant differences among the two institutions are reported regarding the postoperative prescribing and consumption of analgesics, we cannot conclude that our findings apply to other medical centers in these countries.

Another limitation of the study consists in the lack of data regarding pain control after discharge. Based on the results of this study, we plan to investigate whether the type of analgesic (opioid vs non-opioid) prescribed at discharge is influenced by patients’ characteristic or could influence the patients self-reported pain scores when returning to daily activities. In addition, pre-operative and intraoperative pain management could have influenced the results of our study.

Dipyrone was only administered in Israel, which could bias our results towards the remarkably lower opioid and non-opioid consumption in Israel than in the U.S. However, even if the dichotomy introduced some degree of bias in dipyrone administration, this strongly supports our call for the pressing need to consider non-opioid alternatives. Furthermore, we do not intend to advocate for dipyrone administration in the U.S.

Our cohort of patients included adults only. However, as the opioid epidemic in the U.S. can also have cultural roots feeding a more permissive approach towards opioids, the authors believe that a similar study is essential for the pediatric population to evaluate whether excessive opioid exposure starts at a younger age.

## Conclusions

We believe that our findings provide convincing evidence of the permissive practices of postoperative opioid prescription and consumption in adults at HMC (U.S.), compared to SMC (Israel) following similar otolaryngological surgeries. Therefore, we propose a reevaluation of postoperative pain management strategies including clinicians’ education on multimodal analgesia approaches based on institutional preferences and medication availability.

## Data Availability

The datasets used and/or analyzed during the current study is available from the corresponding author on reasonable request.
